# Association of Adiponectin Gene (*ADIPOQ*) rs2241766 Polymorphism with Obesity in Adults: A Meta-Analysis

**DOI:** 10.1371/journal.pone.0095270

**Published:** 2014-04-16

**Authors:** Jingjing Wu, Zheng Liu, Kai Meng, Ling Zhang

**Affiliations:** 1 Department of Epidemiology and Biostatistics, School of Public Health, Capital Medical University, Beijing, China; 2 Beijing Municipal Key Laboratory of Clinical Epidemiology, Beijing, China; 3 Department of Hospital Management, School of Health Administration and Education, Capital Medical University, Beijing, China; Temple University, United States of America

## Abstract

**Background:**

Adiponectin plays an important role in regulating glucose levels and fatty acid oxidation. Multiple studies have assessed the association between rs2241766 polymorphism in the adiponectin *(ADIPOQ)* gene and obesity susceptibility. However, the results are inconsistent and inconclusive. The aim of this meta-analysis was to investigate this association in adults.

**Method:**

Several electronic databases were searched for relevant literature published up to November 2013. Statistical analyses were performed using software Review Manager (Version 5.02) and STATA (Version 10.0). The pooled odds ratios (ORs) and 95% confidence intervals (CIs) were calculated with a random-effects model or a fixed-effect model depending on heterogeneity among studies. Q tests and Egger’s tests were performed to assess heterogeneity and publication bias. Sensitivity analysis was conducted to confirm the reliability and stability of the meta-analysis.

**Results:**

A total of 2,819 obese and 3,024 controls in 18 case-control studies were included in the meta-analysis. The results indicated that compared with TT genotype, the *ADIPOQ*-rs2241766 GG genotype was associated with an increased risk for obesity (OR = 1.39, 95% CI: 1.11–1.73, *P* for heterogeneity = 0.520, *I^2^* = 0%) in overall studies. Whereas, GT genotype was associated with a borderland increased risk for obesity (OR = 1.13, 95% CI: 0.94–1.36, *P* for heterogeneity = 0.006, *I^2^* = 51%). The susceptibility of obesity was increased based on genotypes of TT<GT<GG (*P* for trend = 0.011). Subgroup analysis of different regions revealed that the *ADIPOQ*-rs2241766 GG genotype increased obesity risk in the Chinese studies (OR = 1.54, 95% CI: 1.19–2.00) but not in the non-Chinese studies (OR = 1.02, 95% CI: 0.66–1.58). Similar results were observed in allelic, recessive, and dominant genetic models. There was no significant evidence of publication bias in the overall, Chinese, and non-Chinese studies (*P* = 0.426, *P* = 0.935, and *P* = 0.390, respectively).

**Conclusion:**

The results of this meta-analysis suggest that the *ADIPOQ*-rs2241766 G/T polymorphism might be associated with obesity in Chinese studies but not in non-Chinese studies in adults. Better-designed studies that consider confounding factors and assess larger sample sizes with a focus on *ADIPOQ*-rs2241766G/T polymorphisms and obesity are required in the future.

## Introduction

Overweight/obese status is the fifth leading risk factor for global deaths and is a major global public health problem. Obesity is a medical condition in which excess body fat is accumulated. Adipose tissue is regarded as an endocrine organ that secretes a number of adipocytokines (e.g., adiponectin and tumor necrosis factor-α), which play important roles in the development of obesity, insulin resistance, and their associated complications [1].

Adiponectin is a protein secreted by adipose tissue that was independently discovered by different researchers in the 1990s and named “adipocyte complement-related protein of 30 kDA” (ACRP30) [2], “gelatin-binding protein 28” (GBP28) [3], “AdipoQ” [4], and “adipose most abundant gene transcript 1” (APM1) [5]. This protein is encoded by the adiponectin (*ADIPOQ,* also known as *APM1*) gene, which is comprised of three exons and two introns (17 kb). The *ADIPOQ* gene is located on chromosome 3q27, which has been demonstrated to be a susceptibility locus for obesity by several genome-wide scan studies [6]–[8]. Researchers have reported that adiponectin in plasma can regulate fatty acid oxidation and glucose levels through the phosphorylation and activation of AMP-activated protein kinase (AMPK) [9]. Moreover, adiponectin levels are significantly decreased in patients with obesity, type 2 diabetes mellitus, or coronary artery disease [10], [11]. A genome-wide association study (GWAS) revealed that the *ADIPOQ* gene could explain 6.7% of the phenotypic variance for plasma adiponectin [12]. *ADIPOQ* gene variations were significantly associated with plasma adiponectin levels in several studies [13]–[16], including the rs2241766 G/T polymorphism (*P* = 0.01) [14].

The synonymous single nucleotide polymorphism (SNP) rs2241766 in *ADIPOQ* has been considered to be a candidate SNP for obesity. A number of case-control studies examining the association between *ADIPOQ*-rs2241766 G/T polymorphism and obesity have been published worldwide [17]–[22]. However, there have been inconsistent findings in different populations. Variation in rs2241766 was significantly associated with obesity in studies conducted in Belgium [17] and China [18], [19]. However, conflicting results were published for Swedish [20] and Chinese populations [21], [22]. These discrepancies could be related to racial or regional differences in *ADIPOQ* polymorphism frequency. Therefore, we performed a meta-analysis to examine the association between A*DIPOQ*-rs2241766 G/T polymorphism and obesity in adults.

## Materials and Methods

### Literature Search Strategy

We invited a professional librarian to help us to search literature in the following databases: PubMed, Medline, Embase, ISI Web of Knowledge, Biosis Preview, Ovid, Science Direct, The Cochrane Library, Chinese Wan Fang database, China National Knowledge Infrastructure (CNKI), and Chinese Biomedical Literature Database. Two investigators (JJW and ZL) searched all above databases using appropriate descriptions matching the different retrieval databases. For example, the search strategy for the PubMed database was “(adiponectin or ADIPOQ or APM1 or ADPN) and (rs2241766 or T45G or SNP45 or Gly15Gly or SNP+45 or 45T>G) and (obes* or BMI or (body mass index))” in the title and abstract. The search was limited to human studies. Searching languages included English and Chinese. The literature search was updated to November 2013.

### Inclusion and Exclusion Criteria

The studies included in this meta-analysis were confined to the following criteria: (1) case-control studies focusing on the association between *ADIPOQ*-rs2241766 G/T polymorphism and obesity in adults, (2) clear description of the diagnostic criteria for obesity and sources of subjects, (3) genotype frequencies of rs2241766 in obese and control groups or odds ratio (OR) and 95% confidence interval (CI), and (4) Hardy-Weinberg equilibrium (*HWE*) in the control group.

The following studies were excluded: (1) those that were not designed as case-control studies; (2) reviews, abstracts (without data), comments (without data), and duplications of publications (if there was more than one study with the same population by different investigators or overlapping data by the same authors, we selected the complete articles with the largest number of subjects); (3) studies with control groups that deviated from *HWE*; (4) studies with participants diagnosed with secondary obesity or other serious diseases; or (5) studies that included children.

### Data Extraction

Two researchers (JJW and ZL) independently reviewed all studies to determine whether an individual study could be retained for the meta-analysis and extracted information and data from all included studies. All disagreements were discussed with a third reviewer (KM) until consensus was reached.

The following information was obtained from each study: the last name of the first author, the year of publication, country, source of controls, gender and age of the enrolled subjects, definition of obesity, numbers of genotypes in cases and controls, and *HWE* in each control group. The control source was stratified to population-based (PB) and hospital-based (HB) studies. If necessary data were not reported in the primary paper, we contacted the corresponding authors by e-mail to request the missing data.

### Statistical Analysis

We performed overall and subgroup meta-analyses, the latter were stratified by ethnicity. The pooled ORs and 95% CIs were calculated to measure the strength of the genetic association between *ADIPOQ-*rs2241766 G/T polymorphism and obesity. The significance of the pooled effect size was determined by *Z* tests, and *P*<0.05 was considered statistically significant. The heterogeneity test was analyzed using the *Q*-test and *I^2^* statistic [23]. A *P* value of the *Q*-test >0.10 indicated no heterogeneity among the studies, and the Mantel-Haenszel fixed-effect model could be used as the pooling method. Otherwise the random-effects model was adopted. *I^2^* statistic (between 0% and 100%) documented the percentage of observed study variability due to heterogeneity rather than chance and was used to assess heterogeneity [23]. All above statistical analyses were performed using Review Manager (Version 5.0.2, the Cochrane collaboration). Publication bias was evaluated with funnel plots and Egger’s regression tests [24] with STATA software version 10.0 (STATA Corp., College Station, Texas, USA). Each point in the funnel plots represents a separate study included in the meta-analysis. Both the symmetrical shape of the funnel plots and *P*<0.05 suggested no evidence of publication bias. We calculated the *P*-value of *HWE* for the control group in each study by Chi-square tests, and *P*≥0.01 was considered to adhere to *HWE*.

## Results

### Study Characteristics

**Figure 1:**
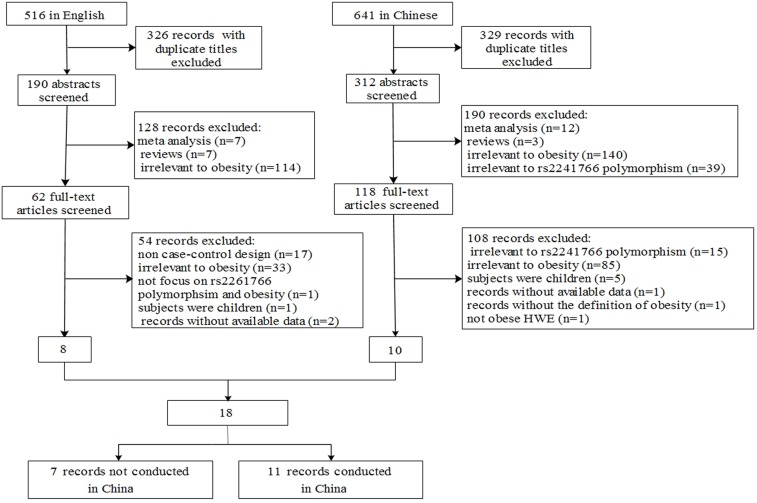
Flow diagram of eligible study selection.

Figure 1 shows the literature search and selection flow chart. A total of 1,157 studies (516 in English and 641 in Chinese) were identified through the database search. After reading the titles and abstracts, 655 studies with duplicate titles and 401 articles that were review articles or assessed unrelated diseases were excluded. The main text of 101 studies was carefully reviewed, and we excluded 83 papers that assessed unrelated polymorphisms, were not case-control designs, were conducted in pediatric populations, did not define obesity, or studied controls that deviated from *HWE*. Finally, 18 relevant studies (1 study published in English was conducted in a Chinese population) that included 2,819 cases and 3,024 controls concerning *ADIPOQ*-rs2241766 G/T polymorphism and obesity were eligible for the meta-analysis [17]–[22], [25]–[36]. Table 1 summarizes the main information of the selected studies, including source of controls, genders, age, definition of obesity, frequencies of genotype and allele in cases and controls, and *HWE*. Overall, 11 studies were carried out in China, and 7 were performed in other countries, which were categorized as “Studies not conducted in China”.

**Table 1:** Characteristics of studies of the association between *ADIPOQ*-rs2241766 G/T polymorphism and obesity.

Author	Year	Country	Source of controls	Gender (male, %)^a^	Age^b^	Definition^c^	GG		GT		TT		G		*HWE* d
							case	control	case	control	case	control	case	control	
Ai ZH [25]	2006	China	PB	129 (62.93)	48.80±10.10	BMI≥25.0	11	7	48	32	57	50	70	44	0.559
Bu RF [26]	2007	China	PB	53 (62.35)	56.53±9.00	BMI≥25.0	2	2	25	10	18	28	29	14	0.396
Chen XY [18]	2012	China	PB	158 (33.55)	45.40±9.01	BMI≥25.0	20	13	132	85	96	125	172	111	0.771
Jin LZ [21]	2004	China	PB	144 (46.75)	53.60±10.69	BMI≥25.0	9	1	41	75	72	107	59	83	0.026
Shi XH [27]	2007	China	PB	162 (56.64)	45.17±5.81	BMI≥28.0	0	11	10	62	13	76	10	84	0.734
Su QJ [28]	2005	China	PB	NA	47.65±11.02	BMI≥25.0	2	3	11	33	17	29	15	39	0.092
Wang CJ [19]	2005	China	HB	100 (46.51)	51.48±8.49	BMI≥25.0	10	5	52	40	41	67	72	50	0.752
Wang SF [29]	2005	China	PB	65 (55.08)	33.56±8.88	BMI≥25.0	6	10	30	32	16	24	42	52	0.901
Wang SJ [22]	2008	China	PB	171 (43.85)	51.00±1.00	BMI≥25.0	15	10	79	70	114	102	108	90	0.654
Wei YL [30]	2007	China	PB	59 (58.42)	53.00±11.00	BMI≥25.0	0	0	10	33	15	43	10	33	0.016
Yan WL [31]	2006	China	PB	273 (55.26)	48.50±9.52	BMI≥28.0	95	72	186	203	201	222	376	347	0.024
Arnaiz-Villena A [32]	2013	Spain	PB	139 (43.17)	NA	M: WC≥88.5	14	6	38	27	124	111	66	39	0.017
						F: WC≥82.5									
Beckers S [17]	2009	Belgium	PB+HB	0 (0.00)	37.28±1.00	BMI≥30.0	5	4	38	24	180	59	48	32	0.450
Bouatia-Naji N [33]	2006	France	PB	NA	NA	BMI≥40.0	14	15	148	144	468	536	179	174	0.155
Boumaiza I [34]	2011	Tunisia	HB	92 (27.96)	45.76±12.10	BMI≥30.0	8	8	48	56	104	105	64	72	0.067
Guzman-Ornelas MO [35]	2012	Mexico	PB	43 (29.66)	37.78±11.16	BMI >30.0	3	6	17	28	37	54	23	40	0.377
Sharma A [36]	2009	America	PB	NA	NA	BMI≥30.0	0	0	5	11	18	49	5	11	0.434
Ukkola O [20]	2003	Sweden	HB	0 (0.00)	45.95±5.50	M: BMI≥34.0F: BMI≥38.0	0	2	13	12	83	82	13	16	0.075

Note: PB: population-based; HB: hospital-based; BMI: body mass index; WC: waist circumference; NA: not available; HWE: Hardy-Weinberg equilibrium; M: male; F: female;

a number and percentage;

b mean ± SD;

c definition of obesity (BMI: kg/m^2^; WC: cm);

d HEW in controls.

### Meta-analysis of all Eligible Studies

**Figure 2:**
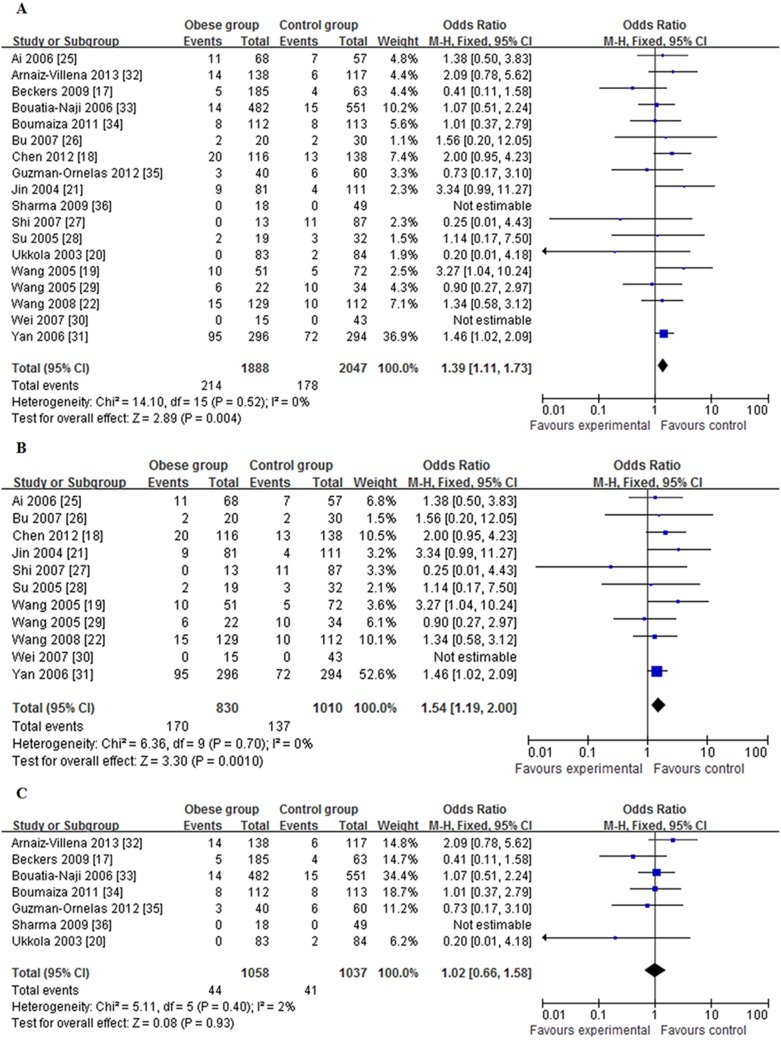
Forest plots regarding the association of *ADIPOQ*-rs2241766 G/T polymorphism with obesity (GG *vs.* TT). (A) in all studies; (B) in Chinese studies; (C) in non-Chinese studies. Studies are listed individually. The OR is presented graphically by a square box to indicate the point estimate and the lines on each side indicate the 95% CI. Box sizes are proportional to inverse-variance weights. This graph is centered by OR = 1 (equivalent to a finding without effect), Points at the right and left of the center line indicate OR>1 and OR<1, respectively.

Overall, no significant heterogeneity was detected between the *ADIPOQ*-rs2241766 G/T polymorphism and obesity risk (*P* for heterogeneity = 0.520, *I^2^* = 0%) in the addictive genetic model (GG *vs.* TT), and a fixed-effect model was employed to assess the combined OR value and 95% CI. The results showed that *ADIPOQ*-rs2241766 GG homozygote status was significantly associated with an increased risk of obesity compared with TT wild-type homozygote, while one mutation allele (GT) could not significantly increase the risk of obesity (GG *vs.* TT: OR = 1.39, 95% CI: 1.11–1.73, Figure 2A, Table 2; GT *vs.* TT: OR = 1.13, 95% CI: 0.94–1.36, *P* for heterogeneity = 0.006, *I^2^* = 51%; *P* for trend  = 0.011). A significant association was also observed in the recessive model (GG *vs.* GT+TT: OR = 1.31, 95% CI: 1.06–1.62), but not in the dominant model (GG+GT *vs.* TT: OR = 1.15, 95% CI: 0.96–1.38) or allele comparison (G *vs.* T: OR = 1.13, 95% CI: 0.98–1.30).

**Table 2:** The meta-analysis results between *ADIPOQ*-rs2241766 G/T polymorphism and obesity in addictive model (GG vs. TT).

Study	No. of studies	Test of association		Effects model	Test of heterogeneity	
		OR(95% CI)	*P* value		*P* value	*I* 2 (%)
In overall studies	18	1.39 (1.11–1.73)	0.004	Fixed	0.520	0
In Chinese studies	11	1.54 (1.19–2.00)	0.001	Fixed	0.700	0
In Chinese studies (exclude studies 27 and 31)^1^	9	1.74 (1.19–2.54)	0.004	Fixed	0.740	0
In non-Chinese studies	7	1.02 (0.66–1.58)	0.930	Fixed	0.400	2
In non-Chinese studies (exclude studies 20, 32 and 33)^2^	4	0.74 (0.36–1.51)	0.410	Fixed	0.580	0
In non-Chinese studies (exclude studies 17 and 20)^3^	5	1.20 (0.75–1.92)	0.460	Fixed	0.650	0

Note: ^1^Meta-analysis in the Chinese studies excluding studies with different diagnostic criteria of obesity;

2 Meta-analysis in the non-Chinese studies excluding studies with different diagnostic criteria of obesity;

3 Meta-analysis in the non-Chinese studies excluding studies that only assessed females.

### Subgroup Analysis

The combined minor allele frequency (MAF) of the G allele was 28.16% in controls among the studies conducted in China and 14.34% in controls among the studies not conducted in China. Distributions of the genotype frequencies in controls were significantly different between the two groups (χ^2^ = 164.84, *P*<0.001). Considering ethnic variations, we performed a subgroup analysis of the studies by population grouping of Chinese studies and non-Chinese studies. Eleven studies including 1,454 obese subjects and 1,685 controls were pooled in the meta-analysis for the Chinese subgroup. The results suggested that compared with TT genotype, subjects carrying the GG genotype had a 1.54-fold higher risk of obesity (95% CI: 1.19–2.00; *P* for heterogeneity = 0.700, *I^2^* = 0%, Figure 2B, Table 2), while subjects carrying one mutation allele (GT) did not had a significantly higher risk of obesity (OR = 1.25, 95% CI: 0.95–1.64; *P* for heterogeneity = 0.005, *I^2^* = 60%; *P* for trend<0.001). No statistically significant association was found in the non-Chinese studies (GG *vs*. TT: OR = 1.02, 95% CI: 0.66–1.58; *P* for heterogeneity = 0.400, *I^2^* = 2%, Figure 2C, Table 2; GT *vs*. TT: OR = 1.02, 95% CI: 0.85–1.23; *P* for heterogeneity = 0.280, *I^2^* = 20%; *P* for trend = 0.896). Similar results were observed in the other three genetic models (data not shown).

### Sensitivity Analysis

In order to assess the stability of the meta-analysis results, sensitivity analyses were performed by omitting the studies with different diagnostic criteria of obesity in Chinese studies [27], [31] and in non-Chinese studies [20], [32]–[33]. We also removed two non-Chinese studies that only assessed females [17], [20] because gender was another confounding factor. The results were not different when these studies were omitted, which suggested that the models were robust (Table 2). Similar results were observed in the other three genetic models (data not shown).

### Publication Bias

**Figure 3:**
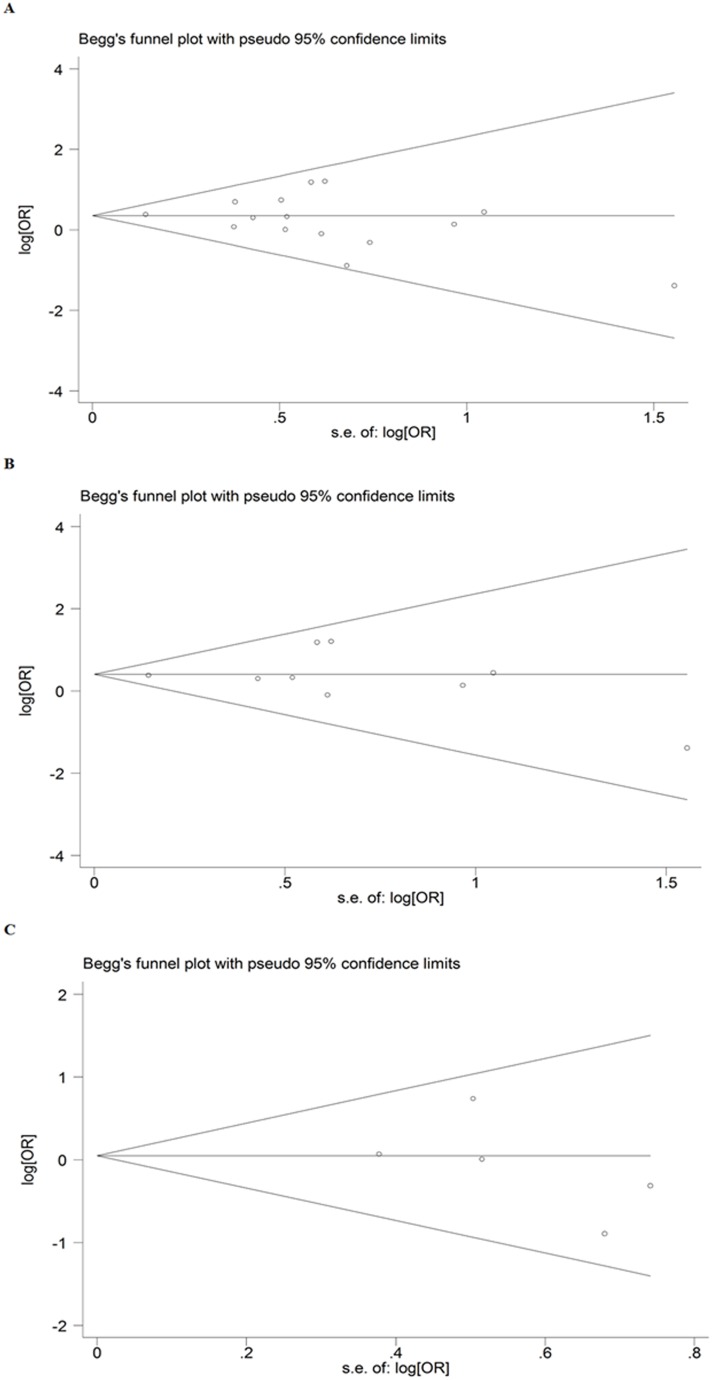
Egger’s funnel plot analyses to detect publication bias (GG *vs.* TT of *ADIPOQ*-rs2241766 G/T polymorphism). (A) All studies, (B) Chinese studies, and (C) non-Chinese studies. Each point represents a separate study included in this meta-analysis. s.e: standardized effect.

Publication bias was examined by funnel plot qualitatively and estimated by Egger’s test quantitatively. The shapes of funnel plots seemed almost symmetrical, suggesting that there was no publication bias for *ADIPOQ*-rs2241766 G/T polymorphism (Figure 3). Egger’s test did not show evidence of publication bias overall (t = −0.82, *P* = 0.426), in Chinese studies (t = −0.08, *P* = 0.935) or in non-Chinese studies (t = −1.00, *P* = 0.390).

## Discussion

The present meta-analysis indicated that the *ADIPOQ*-rs2241766 G/T polymorphism was associated with obesity in overall populations (GG *vs.* TT, OR = 1.39, 95% CI: 1.11–1.73), although one mutation allele (GT) could not significantly increase the risk of obesity. There was a stronger association in the Chinese studies (OR = 1.54, 95% CI: 1.19–2.00) but no relationship in the non-Chinese studies (OR = 1.02, 95% CI: 0.66–1.58). Similar results were observed in the allelic, recessive, and dominant genetic models.

*ADIPOQ* gene was regarded as the only major gene for plasma adiponectin [12]. Adiponectin is secreted primarily by adipose tissue and is thought to mediate increased AMP-activated protein kinase (AMPK) phosphorylation and activity, as well as peroxisome proliferator-activated receptor-a (PPARa) activity, by binding to adiponectin receptors (AdipoR1 and AdipoR2), to activate fatty acid oxidation and glucose uptake both in skeletal and cardiac muscles [37]. Adiponectin-deficient mice exhibit insulin resistance [38], and adiponectin replacement in humans was reported to be a promising method to prevent and/or treat obesity and type 2 diabetes [39]. Interestingly, plasma adiponectin levels were lower in obese individuals than in lean subjects [10] and could be significantly increased by weight loss [40]. These studies suggest that adiponectin is involved in the development of obesity.

Plasma adiponectin levels showed high heritability [41], and a meta-analysis of GWAS determined that only the *ADIPOQ* locus exerted an important effect on plasma adiponectin levels [12]. A variant of *ADIPOQ*-rs2241766 G/T was reported to be associated with adiponectin level [14], [35]. Research showed that this synonymous mutation may affect steady-state mRNA levels by altering RNA splicing or stability [42], suggesting an allele-specific differential expression of adiponectin. The steady-state mRNA levels transcribed by the G allele were higher than those by the T allele in the adipose tissue of heterozygous subjects, and multivariate linear regression analyses with age and gender adjusted showed that the dose of the G allele was associated with a reduction of approximately 1.12 kg/m^2^ in BMI in Taiwan population [42]. Therefore, the speculation that the *ADIPOQ*-rs2241766 G/T polymorphism might be associated with obesity is reasonable.

To our knowledge, the present study is the first meta-analysis to assess the association of rs2241766 polymorphisms with obesity. The G allele appeared to be one of the genetic risk factors for obesity susceptibility in the overall studies especially in the Chinese studies. Adiponectin has also been considered as a marker for metabolic syndrome (MetS). A meta-analysis including 13 studies with 2,684 cases and 2,864 controls in the Chinese population was performed to detect the association of rs2241766 variant with MetS [43], and the results confirmed that the G allele frequency in MetS patients was significantly higher than those of controls (29.8% *vs.* 23.3%, OR = 1.40, *P* = 0.033), which was consistent with our research.

It should be noted that we failed to detect a significant association in the non-Chinese subgroup analysis. The observed significance in the overall studies might be driven only by data from the Chinese studies. The discrepant results in the Chinese studies and non-Chinese studies may be due to differences in the studies’ diagnostic criteria for obesity, genetic backgrounds, gender ratios, environmental effects, and population substructure. The criterion for obesity in most Chinese studies was defined as ≥25 kg/m^2^ [18], [19], [21], [22], [25], [26], [28]–[30], except for two studies that used ≥28 kg/m^2^ [27], [31]. Conversely, most non-Chinese studies defined obesity as ≥30 kg/m^2^ [17], [34]–[36], two studies employed other thresholds [20], [33], and another used waist circumference (WC) to diagnose obesity [32]. Furthermore, two studies were only performed in women [17], [20]. Therefore, we conducted a sensitivity analysis by omitting studies in which obesity was defined by different criteria and studies conducted only in women. The results showed that the pooled ORs and 95% CIs were not obviously influenced by those studies.

In the present study, the G allele frequency in the Chinese studies was significantly higher than in the non-Chinese studies (28.16% *vs.* 14.34%, *P*<0.001), which approximately agreed with the results reported in the NCBI database [44]. Interestingly, the conflicting association between rs2241766 polymorphism and diseases in different ethnicities was also reported in a previous study [45]. A meta-analysis including 5,318 cases and 6,118 controls reported that the G allele was a potential protection factor for breast cancer risk in Caucasians but was not associated with cancer susceptibility in Asians [45]. Similarly, the G allele frequencies of controls were 33.00% in Asians and 16.00% in Caucasians, which were consistent with our findings. Racial heterogeneity might be at least partially attributable to the ethnicity-related distribution of the G allele.

Furthermore, obesity has been reported to be the result of interactions between genetic and environmental factors. We considered that there might be some other causal variants at this locus, and polymorphism, interaction, or linkage disequilibrium affecting these variants could contribute to obesity. Moreover, lifestyle and dietary habits are quite different in the two populations, which may greatly affect the occurrence of obesity. Unfortunately we could not obtain more information about these environmental factors (e.g., eating habits, physical activity, smoking, alcohol intake) to assess the interactions between environmental factors and rs2241766 polymorphism.

Some limitations of the present meta-analysis should be taken into consideration when interpreting the results. Firstly, although we searched multiple databases with the aim to collect all eligible studies, we may have missed publications. The small number of studies included in this meta-analysis also limited the ability to draw more solid conclusions. Secondly, the search language was limited to English and Chinese during the systematic review. Thirdly, this meta-analysis was based on unadjusted ORs and 95% CIs, because we could not obtain the adjusted ORs and 95% CIs from most of the included studies except for one performed in a Tunisian population [34] and another study that defined obesity by WC [32]. No significant association between the rs2241766 polymorphism and obesity was found in either of these studies. Fourth, obesity is a result of the interaction between environmental factors and genetic loads. More than 200 genes have been recognized as obesity candidate genes in association studies. However, we could not address gene-gene or gene-environment interactions due to a lack of related information in the included studies.

In conclusion, this meta-analysis indicated that the *ADIPOQ*-rs2241766 G allele might be associated with increased risk of obesity in adults in the Chinese studies but not in non-Chinese studies. Better designed studies that consider confounding factors in larger sample sizes with a focus on the relationship between *ADIPOQ*-rs2241766G/T polymorphisms and obesity are required to confirm our findings.

## Supporting Information

Checklist S1 **PRISMA Checklist.**
(DOC)
